# (*E*)-3,5-Dimeth­oxy­benzaldehyde oxime

**DOI:** 10.1107/S1600536810038766

**Published:** 2010-10-02

**Authors:** Bin Dong, Yu Zhang, Jin-Zhe Chen

**Affiliations:** a Affliated Hospital of Hebei University, Baoding 071000, People’s Republic of China; b Hebei Xushui County Health Bureau, Baoding 071000, People’s Republic of China

## Abstract

In the title compound, C_9_H_11_NO_3_, the oxime grouping is twisted by 12.68 (6)° with respect to the dimethoxyl­benzene ring. In the crystal, mol­ecules are linked into an infinite [100] chain *via* O—H⋯N hydrogen bonds, instead of the more common oxime packing motif of dimers with an *R*_2_^2^(6) graph-set motif.

## Related literature

For backgroud to oximes as therapeutic agents, see: Marrs *et al.* (2006 ▶); Jokanovic *et al.* (2009 ▶). For related structures, see: Bao (2008 ▶); Abbas *et al.* (2010 ▶). For graph-set theory, see: Etter *et al.* (1990 ▶); Bernstein *et al.* (1995 ▶).
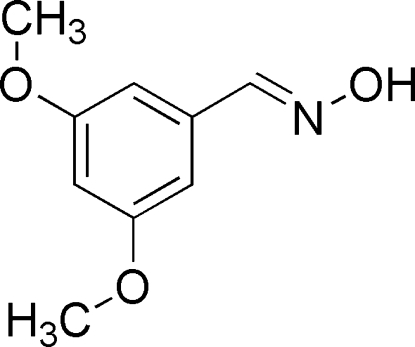


## Experimental

### 

#### Crystal data

C_9_H_11_NO_3_
*M* *_r_* = 181.19
Orthorhombic, 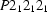

*a* = 4.4027 (9) Å
*b* = 13.800 (3) Å
*c* = 14.300 (3) Å
*V* = 868.9 (3) Å^3^
*Z* = 4
Mo *K*α radiation
μ = 0.11 mm^−1^
*T* = 113 K
0.20 × 0.18 × 0.10 mm

#### Data collection

Rigaku Saturn CCD area-detector diffractometer
Absorption correction: multi-scan (*CrystalClear*; Rigaku/MSC, 2005 ▶) *T*_min_ = 0.979, *T*_max_ = 0.990
7173 measured reflections
1239 independent reflections
1115 reflections with *I* > 2σ(*I*)
*R*_int_ = 0.036

#### Refinement

*R*[*F* ^2^ > 2σ(*F* ^2^)] = 0.030
*wR*(*F* ^2^) = 0.081
*S* = 1.08
1239 reflections
124 parameters
H atoms treated by a mixture of independent and constrained refinement
Δρ_max_ = 0.22 e Å^−3^
Δρ_min_ = −0.17 e Å^−3^

### 

Data collection: *CrystalClear* (Rigaku/MSC, 2005 ▶); cell refinement: *CrystalClear*; data reduction: *CrystalClear*; program(s) used to solve structure: *SHELXS97* (Sheldrick, 2008 ▶); program(s) used to refine structure: *SHELXL97* (Sheldrick, 2008 ▶); molecular graphics: *SHELXTL* (Sheldrick, 2008 ▶); software used to prepare material for publication: *SHELXL97*.

## Supplementary Material

Crystal structure: contains datablocks I, global. DOI: 10.1107/S1600536810038766/hb5656sup1.cif

Structure factors: contains datablocks I. DOI: 10.1107/S1600536810038766/hb5656Isup2.hkl

Additional supplementary materials:  crystallographic information; 3D view; checkCIF report

## Figures and Tables

**Table 1:** Hydrogen-bond geometry (Å, °)

*D*—H⋯*A*	*D*—H	H⋯*A*	*D*⋯*A*	*D*—H⋯*A*
O3—H3⋯N1^i^	0.916 (19)	1.90 (2)	2.7970 (17)	166.5 (19)

Symmetry code: (i) 
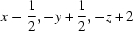
.

**Fig. 1.**
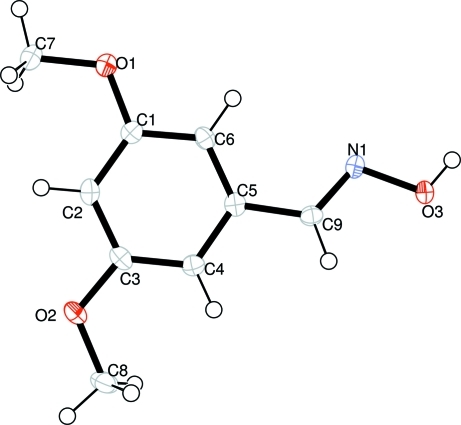
The molecule of (I) showing displacement ellipsoids drawn at the 50% probability level.

**Fig. 2.**
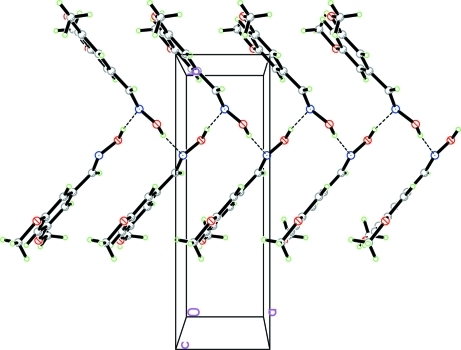
The infinite chain formed via O—H···N down the a axis.

## supplementary crystallographic information

### Comment

Oximes are an therapeutic agent in organophosphorus poisoning (Marrs *et al.*, 2006; Jokanovic *et al.*, 2009). As part of our interest in the study of oxime derivatives, we herein report the crystal structure of the title compound (I).

In the crystal structure of the title compound, Fig. 1, the oxime moiety has an E configuration [C5—C9—N1—O3= 178.22 (11)°] and is twisted with respect to the dimethoxylbenzene ring by 12.68 (6)°. Molecules are linked to form an infinite chain down the *a* axis *via* O—H···N hydrogen bonds (Fig. 2 and Table 1), which differates from the reported *R*_2_^2^(6) graph-set motif (Etter *et al.*, 1990; Bernstein *et al.*, 1995; Bao, 2008; Abbas *et al.*, 2010).

### Experimental

To a solution of 3,4-dimethoxylbenzaldehyde (0.95 g, 5 mmol) in 25 ml e thanol, hydroxylamine hydrochloride (0.42 g, 6 mmol) and aqueous sodium hydroxide (0.24 g, 6 mmol) were added and the mixture was heated under reflux until completion of the reaction. The reaction mixture was concentrated and water added. The precipitate was collected by filtration, washed with water and dried under vaccu. Colourless blocks of (I) were grown out *via* recrystallization from ethanol.

### Refinement

All H atoms were placed in calculated position and treated as riding on their parent atoms with C—H = 0.93 and 0.97Å or O—H =0.82 Å with *U*_iso_(H) = 1.2 *U*_eq_(C) for aromatic H atoms, or 1.5*U*_eq_ (O and C) for hydroxyl H and methyl H atom].

### Crystal data

**Table:**

C_9_H_11_NO_3_	*F*(000) = 384
*M**_r_* = 181.19	*D*_x_ = 1.385 Mg m^−^^3^
Orthorhombic, *P*2_1_2_1_2_1_	Mo *K*α radiation, λ = 0.71073 Å
Hall symbol: P 2ac 2ab	Cell parameters from 3117 reflections
*a* = 4.4027 (9) Å	θ = 2.1–27.9°
*b* = 13.800 (3) Å	µ = 0.11 mm^−^^1^
*c* = 14.300 (3) Å	*T* = 113 K
*V* = 868.9 (3) Å^3^	Block, colorless
*Z* = 4	0.20 × 0.18 × 0.10 mm

### Data collection

**Table:**

Rigaku Saturn CCD area-detector diffractometer	1239 independent reflections
Radiation source: rotating anode	1115 reflections with *I* > 2σ(*I*)
multilayer	*R*_int_ = 0.036
Detector resolution: 7.31 pixels mm^-1^	θ_max_ = 27.9°, θ_min_ = 2.1°
ω and φ scans	*h* = −5→5
Absorption correction: multi-scan (*CrystalClear*; Rigaku/MSC, 2005)	*k* = −18→12
*T*_min_ = 0.979, *T*_max_ = 0.990	*l* = −18→18
7173 measured reflections	

### Refinement

**Table:**

Refinement on *F*^2^	Secondary atom site location: difference Fourier map
Least-squares matrix: full	Hydrogen site location: inferred from neighbouring sites
*R*[*F*^2^ > 2σ(*F*^2^)] = 0.030	H atoms treated by a mixture of independent and constrained refinement
*wR*(*F*^2^) = 0.081	*w* = 1/[σ^2^(*F*_o_^2^) + (0.0566*P*)^2^ + 0.0067*P*] where *P* = (*F*_o_^2^ + 2*F*_c_^2^)/3
*S* = 1.08	(Δ/σ)_max_ = 0.001
1239 reflections	Δρ_max_ = 0.22 e Å^−^^3^
124 parameters	Δρ_min_ = −0.16 e Å^−^^3^
0 restraints	Extinction correction: *SHELXL97* (Sheldrick, 2008), Fc^*^=kFc[1+0.001xFc^2^λ^3^/sin(2θ)]^-1/4^
Primary atom site location: structure-invariant direct methods	Extinction coefficient: 0.135 (12)

### Special details

**Table:**

Geometry. All e.s.d.'s (except the e.s.d. in the dihedral angle between two l.s. planes) are estimated using the full covariance matrix. The cell e.s.d.'s are taken into account individually in the estimation of e.s.d.'s in distances, angles and torsion angles; correlations between e.s.d.'s in cell parameters are only used when they are defined by crystal symmetry. An approximate (isotropic) treatment of cell e.s.d.'s is used for estimating e.s.d.'s involving l.s. planes.
Refinement. Refinement of *F*^2^ against ALL reflections. The weighted *R*-factor *wR* and goodness of fit *S* are based on *F*^2^, conventional *R*-factors *R* are based on *F*, with *F* set to zero for negative *F*^2^. The threshold expression of *F*^2^ > σ(*F*^2^) is used only for calculating *R*-factors(gt) *etc*. and is not relevant to the choice of reflections for refinement. *R*-factors based on *F*^2^ are statistically about twice as large as those based on *F*, and *R*- factors based on ALL data will be even larger.

### Fractional atomic coordinates and isotropic or equivalent isotropic displacement parameters (Å^2^)

**Table:**

	*x*	*y*	*z*	*U*_iso_*/*U*_eq_	
O1	1.1440 (3)	0.57833 (8)	1.13939 (7)	0.0215 (3)	
O2	1.2719 (3)	0.66671 (7)	0.81997 (7)	0.0212 (3)	
O3	0.2763 (3)	0.27744 (7)	0.90865 (7)	0.0195 (3)	
H3	0.211 (5)	0.2351 (13)	0.9537 (13)	0.029*	
N1	0.4898 (3)	0.33604 (8)	0.95540 (9)	0.0159 (3)	
C1	1.0843 (4)	0.56661 (10)	1.04608 (10)	0.0170 (3)	
C2	1.2075 (4)	0.62501 (10)	0.97646 (10)	0.0179 (3)	
H2	1.3404	0.6767	0.9923	0.021*	
C3	1.1337 (4)	0.60688 (10)	0.88299 (10)	0.0169 (3)	
C4	0.9316 (4)	0.53398 (10)	0.85910 (10)	0.0168 (3)	
H4	0.8775	0.5233	0.7956	0.020*	
C5	0.8078 (4)	0.47596 (10)	0.93064 (10)	0.0156 (3)	
C6	0.8833 (4)	0.49179 (10)	1.02358 (10)	0.0167 (3)	
H6	0.7994	0.4522	1.0714	0.020*	
C7	1.3439 (4)	0.65620 (10)	1.16453 (11)	0.0221 (4)	
H7A	1.5396	0.6476	1.1329	0.033*	
H7B	1.3752	0.6561	1.2324	0.033*	
H7C	1.2536	0.7180	1.1455	0.033*	
C8	1.2008 (4)	0.65223 (11)	0.72320 (10)	0.0255 (4)	
H8A	1.2574	0.5862	0.7048	0.038*	
H8B	1.3140	0.6990	0.6852	0.038*	
H8C	0.9824	0.6616	0.7135	0.038*	
C9	0.5886 (3)	0.40214 (10)	0.90072 (10)	0.0161 (3)	
H9	0.5170	0.4035	0.8381	0.019*	

### Atomic displacement parameters (Å^2^)

**Table:**

	*U*^11^	*U*^22^	*U*^33^	*U*^12^	*U*^13^	*U*^23^
O1	0.0254 (6)	0.0209 (5)	0.0180 (5)	−0.0057 (5)	−0.0022 (5)	−0.0001 (4)
O2	0.0242 (6)	0.0191 (5)	0.0204 (5)	−0.0043 (4)	0.0028 (5)	0.0047 (4)
O3	0.0208 (6)	0.0195 (5)	0.0182 (5)	−0.0073 (5)	−0.0012 (5)	−0.0006 (4)
N1	0.0136 (6)	0.0151 (6)	0.0188 (6)	−0.0007 (5)	−0.0006 (6)	−0.0019 (4)
C1	0.0169 (7)	0.0151 (7)	0.0189 (7)	0.0014 (6)	−0.0015 (6)	−0.0009 (5)
C2	0.0161 (7)	0.0137 (6)	0.0238 (7)	−0.0007 (6)	−0.0006 (6)	−0.0007 (5)
C3	0.0156 (7)	0.0135 (7)	0.0218 (7)	0.0024 (6)	0.0032 (6)	0.0038 (5)
C4	0.0175 (7)	0.0163 (7)	0.0166 (7)	0.0015 (6)	0.0001 (6)	0.0009 (5)
C5	0.0132 (7)	0.0134 (6)	0.0203 (7)	0.0018 (6)	−0.0003 (6)	0.0002 (5)
C6	0.0162 (7)	0.0149 (7)	0.0188 (7)	−0.0005 (6)	0.0008 (6)	0.0020 (5)
C7	0.0230 (8)	0.0214 (8)	0.0218 (8)	−0.0030 (6)	−0.0016 (7)	−0.0046 (6)
C8	0.0334 (10)	0.0252 (8)	0.0179 (8)	−0.0010 (7)	0.0049 (7)	0.0048 (6)
C9	0.0157 (7)	0.0171 (7)	0.0156 (7)	0.0010 (6)	−0.0014 (6)	0.0000 (5)

### Geometric parameters (Å, °)

**Table:**

O1—C1	1.3697 (18)	C4—C5	1.4089 (19)
O1—C7	1.4349 (19)	C4—H4	0.9500
O2—C3	1.3653 (17)	C5—C6	1.387 (2)
O2—C8	1.4328 (17)	C5—C9	1.467 (2)
O3—N1	1.4087 (15)	C6—H6	0.9500
O3—H3	0.916 (19)	C7—H7A	0.9800
N1—C9	1.2777 (18)	C7—H7B	0.9800
C1—C2	1.391 (2)	C7—H7C	0.9800
C1—C6	1.397 (2)	C8—H8A	0.9800
C2—C3	1.398 (2)	C8—H8B	0.9800
C2—H2	0.9500	C8—H8C	0.9800
C3—C4	1.386 (2)	C9—H9	0.9500
			
C1—O1—C7	116.76 (12)	C5—C6—C1	119.26 (14)
C3—O2—C8	117.12 (12)	C5—C6—H6	120.4
N1—O3—H3	103.9 (12)	C1—C6—H6	120.4
C9—N1—O3	110.28 (12)	O1—C7—H7A	109.5
O1—C1—C2	123.64 (14)	O1—C7—H7B	109.5
O1—C1—C6	115.67 (13)	H7A—C7—H7B	109.5
C2—C1—C6	120.68 (14)	O1—C7—H7C	109.5
C1—C2—C3	119.34 (14)	H7A—C7—H7C	109.5
C1—C2—H2	120.3	H7B—C7—H7C	109.5
C3—C2—H2	120.3	O2—C8—H8A	109.5
O2—C3—C4	124.22 (13)	O2—C8—H8B	109.5
O2—C3—C2	114.79 (13)	H8A—C8—H8B	109.5
C4—C3—C2	120.98 (13)	O2—C8—H8C	109.5
C3—C4—C5	118.82 (14)	H8A—C8—H8C	109.5
C3—C4—H4	120.6	H8B—C8—H8C	109.5
C5—C4—H4	120.6	N1—C9—C5	122.77 (13)
C6—C5—C4	120.89 (14)	N1—C9—H9	118.6
C6—C5—C9	123.12 (13)	C5—C9—H9	118.6
C4—C5—C9	115.94 (13)		
			
C7—O1—C1—C2	0.4 (2)	C3—C4—C5—C6	0.7 (2)
C7—O1—C1—C6	−178.53 (13)	C3—C4—C5—C9	178.22 (13)
O1—C1—C2—C3	179.67 (14)	C4—C5—C6—C1	0.2 (2)
C6—C1—C2—C3	−1.4 (2)	C9—C5—C6—C1	−177.15 (13)
C8—O2—C3—C4	0.0 (2)	O1—C1—C6—C5	179.15 (14)
C8—O2—C3—C2	−179.10 (14)	C2—C1—C6—C5	0.2 (2)
C1—C2—C3—O2	−178.50 (13)	O3—N1—C9—C5	178.28 (12)
C1—C2—C3—C4	2.3 (2)	C6—C5—C9—N1	−12.4 (2)
O2—C3—C4—C5	178.97 (14)	C4—C5—C9—N1	170.15 (14)
C2—C3—C4—C5	−2.0 (2)		

### Hydrogen-bond geometry (Å, °)

**Table:**

*D*—H···*A*	*D*—H	H···*A*	*D*···*A*	*D*—H···*A*
O3—H3···N1^i^	0.916 (19)	1.90 (2)	2.7970 (17)	166.5 (19)

Symmetry codes: (i) *x*−1/2, −*y*+1/2, −*z*+2.

## References

[bb1] Abbas, A., Hussain, S., Hafeez, N., Badshah, A., Hasan, A. & Lo, K. M. (2010). *Acta Cryst.* E**66**, o1130. 10.1107/S1600536810013978 PMC2979095 21579179

[bb2] Bao, F.-Y. (2008). *Acta Cryst.* E**64**, o2134. 10.1107/S1600536808033217 PMC2959647 21580995

[bb3] Bernstein, J., Davis, R. E., Shimoni, L. & Chang, N. L. (1995). *Angew. Chem. Int. Ed. Engl.* **34**, 1555–1573.

[bb4] Etter, M. C., MacDonald, J. C. & Bernstein, J. (1990). *Acta Cryst.* B**46**, 256–262. 10.1107/s0108768189012929 2344397

[bb6] Jokanovic, M. & Prostran, M. (2009). *Curr. Med. Chem.* **16**, 2177–2188. 10.2174/092986709788612729 19519385

[bb7] Marrs, T. C., Rice, P. & Vale, J. A. (2006). *Toxicol. Rev.* **25**, 297–323. 10.2165/00139709-200625040-00009 17288500

[bb8] Rigaku/MSC (2005). *CrystalClear* and *CrystalStructure* Rigaku Corporation, Tokyo, Japan.

[bb9] Sheldrick, G. M. (2008). *Acta Cryst.* A**64**, 112–122. 10.1107/S0108767307043930 18156677

